# Comparison of the efficacy of 5% versus 8% acetic acid concentrations for detecting premalignant and malignant lesions in colposcopy

**DOI:** 10.1097/MD.0000000000036341

**Published:** 2023-12-15

**Authors:** Alpay Yilmaz, Aysegul Gulbahar, Serhat Sen

**Affiliations:** a Department of Obstetrics and Gynecology, Gynecologic Oncology Surgery, Izmir Katip Celebi University, Ataturk Training and Research Hospital, Izmir, Turkey; b Department of Obstetrics and Gynecology, Izmir Katip Celebi University, Ataturk Training and Research Hospital, Izmir, Turkey; c Department of Gynecologic Oncology, Istinye University Medical Park Hospital, Istanbul, Turkey.

**Keywords:** acetic acid, cervical cancer, colposcopic surgery, human papilloma virus

## Abstract

Although preventable; cervical cancer remains a significant cause of morbidity and mortality worldwide, especially in developing countries; thus, early diagnosis and treatment are essential to prevent its development into invasive cancer. Based on the screening results, diagnostic colposcopy was performed to evaluate women with abnormal Papinocalaou test results, high-risk human papillomavirus DNA positivity, or suspected cervical cancer. Therefore, this study aimed to determine the optimal acetic acid concentration (5% or 8%) for detecting cervical precancerous/cancerous lesions during colposcopy evaluation. This study included 607 patients admitted to our obstetrics and gynecology clinic. The medical records of the patients were obtained from the colposcopy registry in the hospital information system and retrospectively analyzed. The cases were divided into 2 groups according to the acetic acid concentrations (5% and 8%) used during colposcopy and examined. The duration of acetic acid application was 2 to 4 minutes. The probability of developing ≥ CIN2 was low in patients with negative for intraepithelial lesion or malignancy smear results in both groups, but increased in the high-grade squamous intraepithelial lesions/atypical squamous cells group with the 8% acetic acid concentration (*P* < .0001; *P* = .012). The probabilities of pathological detection of ≥ CIN2 in the 5% and 8% acetic acid groups were 17.3% and 46.6%, respectively (*P* < .0001). The enhancement of the efficiency of colposcopy should focus on improving the detectability of precancerous lesions. Given that this study compared the effectiveness of acetic acid concentration in colposcopy diagnostics, it can be considered a leading study in this field.

## 1. Introduction

Although preventable, cervical cancer (CC) remains a significant cause of morbidity and mortality worldwide, especially in developing countries. CC is the second most common type of cancer (15.7 per 100,000 women) in low-income countries and ranks third in cancer deaths (100.000’de 8.3).^[1,2]^ CC is primarily caused by infection with one or more persistent or chronic “high-risk” (or oncogenic) types of human papillomavirus (HPV).^[3]^ CC is particularly associated with HPV types 16 and 18 and is largely preventable; primary and secondary prevention involves vaccination against HPV and population-based screening, respectively.^[4]^

Early diagnosis and treatment are essential to prevent the disease from developing into invasive cancer. The American Cancer Society, Society of Gynecologic Oncology, and European guidelines for quality assurance in CC screening suggest DNA-HPV testing as the best strategy for primary screening.^[5,6]^ The American Society for Colposcopy and Cervical Pathology guidelines suggest screening with Co-Test HPV DNA and smear for women aged 30 to 64 years.^[7]^ Based on screening results, diagnostic colposcopy is performed to evaluate women with abnormal Papinocalau test results, high-risk HPV DNA positivity, or suspected CC. Colposcopy is a procedure used to examine the cervix, vagina, and vulva; it was first used as a screening tool for CC in 1925. Colposcopy differs significantly depending on the performer; therefore, the American Society for Colposcopy and Cervical Pathology has published standardization guidelines for its performance. Thus, the standard colposcopy biopsy procedure is performed after applying 3% to 5% acetic acid to the cervix.^[8,9]^

This study aimed to determine the optimal acetic acid concentration (5% or 8%) for detecting cervical precancerous or cancerous lesions during colposcopy evaluations.

## 2. Materials and methods

### 2.1. Patients

This study included 607 patients that are admitted to the Obstetrics and Gynecology Clinic of Our Hospital, between 2017 and 2019, and underwent colposcopy biopsy. The medical records of the patients were obtained from the colposcopy registry in the hospital information system and retrospectively analyzed. 205 of these patients admitted for checkups 1 year after colposcopy and their results have also been retrospectively analyzed.

### 2.2. Inclusion and exclusion criteria

The inclusion criteria were patients who underwent colposcopy and from whom biopsies were obtained depending on the result of high-risk (oncogenic) HPV and/or pathological cytology. The exclusion criteria were patients with a history of preinvasive or invasive cervical lesions, who had a history of gynecological cancer, and who were pregnant.

### 2.3. Cytological analysis

Cytological analysis was performed by histopathologically staining with Papinocalau stain and evaluation using the bethesda system. HPV-positive patients admitted were divided into “HPV 16 and/or 18” and “HPV Other” groups according to high-risk HPV types and were evaluated.

### 2.4. Colposcopy procedure

The colposcopy procedure was conducted using a colposcopy device (MDS 3300 HD/ALARIS TURKEY) and a camera system (1/4 inch Sony Super HAD color CCD camera). An acetowhite appearance, punctuation, mosaic pattern, and atypical vascularization were considered abnormal findings. Biopsies were obtained from 4 quadrants and suspected lesions in patients who underwent colposcopy by physicians working in our clinic who are experts in the field of gyneco-oncology. The cases were divided into 2 groups according to the acetic acid concentrations (5% and 8%) used during colposcopy and examined. The duration of acetic acid application was 2 to 4 minutes for 1 time. The pathological results after colposcopy biopsy were grouped according to the World Health Organization classification. One year after the colposcopy procedure, 205 patients admitted to the clinic for checkups received a control cervicovaginal smear (CVS).

### 2.5. Ethics statement

Our institutional review board granted approval for the study (No. 134/Date:27.03.2019). Due to the study retrospective nature, the need for formal informed consent was waived. The 1964 Helsinki Declaration of Principles served as the guide for this study conduct.

### 2.6. Statistical analysis

Statistical Package for the Social Sciences 20.0 was used to analyze the data (Armonk, NY, IBM Corp.). The Kolmogorov-Smirnov test was used to check for normality in continuous variables. Age-related data that is normally distributed is shown as mean standard deviation (minimum-maximum). Categorical data are presented as counts and percentages and were analyzed using the chi-square test or Fisher exact test. A *P* value < .05 was considered statistically significant.

## 3. Results

### 3.1. HPV, CVS, colposcopy, and control CVS results

The average age of the 607 participants in this study was 44.9 ± 9.6 (Min-Max/30–74 ages). The result of HPV culture revealed that HPV16, HPV18, or both were positive in 351 (57.8%) patients. The “HPV Other” group comprised 256 patients (42.2%). The first CVS results of the patients before colposcopy were negative for intraepithelial lesion or malignancy (NILM) smear findings, 455 (75%); atypical squamous cells (ASC-H) of undetermined significance, 77 (12.7%); low-grade squamous intraepithelial lesions (LSIL), 45 (7.4%); high-grade squamous intraepithelial lesions (HSIL), 15 (2.5%); and ASC-H, 15 (2.5%). Colposcopy results were as follows: no dysplasia, 434 (71.5%); LSIL, 51 (8.4%); HSIL, 118 (19.4%); and cancer, 4 (0.4%). Of these, 549 (90.4%) and 58 (9.6%) patients underwent colposcopy using 5% and 8% acetic acid solution, respectively. The CVS results of the 205 patients admitted for checkups 1 year after colposcopy showed smear findings NILM; smear findings, 191 (92.7%); ASC-H of undetermined significance, 7 (3.4%); LSIL, 2; (1.1%); HSIL, 3 (1.7%); and ASC-H, 2 (1.1%).

**Figure 1.**
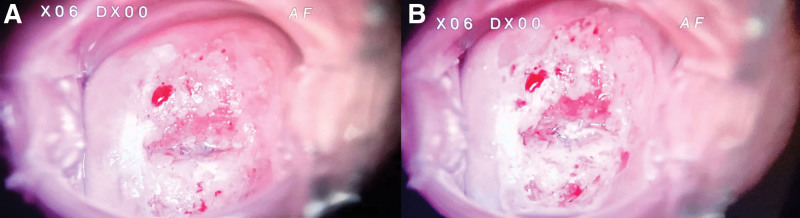
The colposcopic images of the cervix after 5% and 8% acetic acid application. Footnote: 5% acetic acid application (A), 8% acetic acid application (B).

5% and 8% acetic acid application in colposcopy of the cervix are shown in Figure 1 (see Fig. 1, http://links.lww.com/MD/K991, Supplemental Digital Content, which illustrates 5% and 8% acetic acid application in colposcopy of the cervix), respectively. It is observed that the lesions are more prominent after the application of 8% acetic acid.

The results of CVS, HPV, and colposcopy according to the 5% and 8% acetic acid groups of patients and the control CVS of patients admitted for a control smear 1 year after colposcopy are shown in Table 1.

**Table 1:** The distribution of CVS, HPV, colposcopy, and 1-year follow-up smear results between groups.

		5% acetic acid		8% acetic acid	
		N	(%)	n	(%)
HPV	HPV 16–18	316	57.6	35	60.3
	Other	233	42.4	23	39.7
First CVS	NILM	427	77.8	28	48.3
	ASC-US	65	11.8	12	20.7
	LSIL	36	6.6	9	15.5
	HSIL	11	2	4	6.9
	ASC-H	10	1.8	5	8.6
Colposcopic biopsy	No dysplasia	409	74.5	25	43.1
	LSIL	45	8.2	6	10.3
	HSIL	91	16.6	27	46.6
	Cancer	4	0.7	0	0
CVS after colposcopy	NILM	164	92.7	27	96.4
	ASC-US	6	3.4	1	3.6
	LSIL	2	1.1	0	0
	HSIL	3	1.7	0	0
	ASC-H	2	1.1	0	0

ASC-H = atypical squamous cells, ASC-US = atypical squamous cells of undetermined significance, CVS = cervicovaginal smear, HPV = human papillomavirus, HSIL = high-grade squamous intraepithelial lesions, LSIL = low-grade squamous intraepithelial lesions, NILM = negative for intraepithelial lesion or malignancy.

### 3.2. Comparative and statistical analysis of CVS results between groups

The comparative analysis of the CVS results between the groups of colposcopy biopsy results revealed that both groups were statistically significant (Table 2). The probability of developing ≥ CIN2 was low in patients with NILM smear results in both groups but increased in the HSIL/ASC-H group with the 8% acetic acid concentration (*P* < .0001; *P* = .012). No statistical difference was observed when the results of the colposcopy biopsy were compared with those of control CVS obtained 1 year after colposcopy between groups (Table 3). The relationship between the colposcopy biopsy results and the acetic acid concentration showed statistical significance between the groups. The probability of pathological detection of ≥ CIN2 in the 5% and 8% acetic acid group was 17.3% and 46.6%, respectively (*P* < .0001) (Table 4).

**Table 2:** Comparison of colposcopic biopsy groups according to CVS results within acetic acid groups.

Acetic acid concentration	CVS	Colposcopic biopsy		*P*
		Normal- CIN1 n (%)	≥CIN2 n (%)	
5% acetic acid	NILM	358 (83.8)	69 (16.2)	<.0001
	ASC-US/LSIL	88 (87.1)	13 (12.9)	
	HSIL/ASC-H	8 (38.1)	13 (61.9)	
8% acetic acid	NILM	19 (67.9)	9 (32.1)	.012
	ASC-US/LSIL	11 (52.4)	10 (47.6)	
	HSIL/ASC-H	1 (11.1)	8 (88.9)	

ASC-H = atypical squamous cells, ASC-US = atypical squamous cells of undetermined significance, CIN = cervical intraepithelial neoplasia, CVS = cervicovaginal smear, HSIL = high-grade squamous intraepithelial lesions, LSIL = low-grade squamous intraepithelial lesions, NILM = negative for intraepithelial lesion or malignancy.

**Table 3:** Comparison of colposcopic biopsy groups according to first CVS after colposcopy results within acetic acid groups.

Acetic acid concentration	First CVS after colposcopy	Colposcopic biopsy		
		Normal-CIN1 n (%)	≥CIN2 n (%)	*P*
5% acetic acid	NILM	149 (92.5)	15 (93.8)	.668
	Pathological	12 (7.5)	1 (6.2)	
8% acetic acid	NILM	21 (95.5)	6 (100)	.786
	Pathological	1 (4.5)	0 (0)	

CIN = cervical intraepithelial neoplasia, CVS = cervicovaginal smear, NILM = negative for intraepithelial lesion or malignancy.

**Table 4:** The relationship between the colposcopy biopsy results and the acetic acid groups.

		Acetic acid concentration		*P*
		5% acetic acid n (%)	8% acetic acid n (%)	
Colposcopic biopsy	Normal-CIN1	454 (82.7)	31 (53.4)	.0001
	≥CIN2	95 (17.3)	27 (46.6)	
First CVS after colposcopy	NILM	164 (92.7)	27 (96.4)	.403
	Pathological	13 (7.3)	1 (3.6)	

CIN = cervical intraepithelial neoplasia, CVS = cervicovaginal smear, NILM = negative for intraepithelial lesion or malignancy.

## 4. Discussion

Colposcopy is a cornerstone procedure for screening for CC and its precursor lesions. However, the effectiveness of colposcopy in detecting CIN2 + lesions is 30% to 70%,^[10–12]^which is not at the desired level. Although the World Health Organization has defined the colposcopy method as optimal, many studies have been designed to determine the most appropriate method to enhance diagnostic power. The main focus of these studies was to determine ‘the application time of acetic acid and whether blinding or multiple biopsies should be performed.^[13,14]^

These studies indicated that the optimal acetic acid concentration range was 3% to 5%, and designed experiments were conducted within this concentration range.^[15]^ However, no study has examined the optimal range of acetic acid concentration. Supposing the 5% concentration was considered optimal in this study, some difficulties may have ensued, such as inaccuracy in the preparation of the solution during routine clinical practice and loss of concentration due to prolonged dilute retention of the solution. Therefore, the diagnostic power of acetic acid may have been significantly reduced because it might have remained below the optimal concentration threshold. Another critical issue is the validity of considering a concentration range of 3% to 5% as optimal; also, can the success of colposcopy increase when a concentration above 5% is used? No studies have been conducted on this topic. The 8% concentration range in this study was determined because the procedure was unintentionally performed with an 8% concentration while obtaining a 5% dilution in several cases in our clinic. This range was set as the upper limit as there were no side effects detected. As a preliminary assessment, a significant increase was observed in the brightness and limits of existing lesions after applying acetic acid at an 8% dilution concentration to the area; an acetowhite area involvement due to an increase in concentration was detected. Consequently, this study compared the 8% and 5% acetic acid concentrations and examined their difference in detecting high-grade lesions. In the cases where colposcopy was referred due to HPV positivity and/or cytological abnormality, the histopathological detection rate of HSIL was significantly higher in the 8% acetic acid group than in the 5% group (46.6% vs 17.3%). Additionally, the CIN2 + detection rate in cases with normal pre-colposcopy cytology was higher in the 8% acetic acid group than in the 5% group (16.2% vs 32.1%). Similarly, in cases with ASC-US/LSIL or ASC-H/HSIL cytology before colposcopy, a higher rate of HSIL was detected in the 8% acetic acid group. These clinically significant differences indicated that some lesions could be bypassed with 5% acetic acid, suggesting the possibility that they can be captured at higher concentrations, such as 8%.

Supposing the hypothesis that lesions can be captured more by 8% acetic acid is accurate, fewer abnormalities should have been detected in the 5% acetic acid group in the cytological evaluation performed a year after colposcopy. However, in the CVS data obtained a year after colposcopy, pathological cytology was detected in 1/28 (3.5%) and 13/177 (7.3%) cases in the 8% and 5% acetic acid groups, respectively. These rates appeared clinically significant; however, they were not statistically significant, possibly due to the limited number of cases.

The major limitation of this study was the relatively small number of cases (549 and 58 cases in the 5% and 8% acetic acid group). Another limitation was that the cytological distributions of the groups before colposcopy were dissimilar. The regular cytology rate in the 5% group was 427/549 (77.7%), whereas that in the 8% group was 28/58 (48.7%). The distribution of HPV types in both groups was similar; however, the higher cytological anomaly rate in the 8% group may have caused the detection of a higher proportion of CIN2 + in this group. Aforementioned, our study is based on performing with an 8% concentration unintentionally and this resulted to limited data on 8% group, resulting the conclusions to be evaluated as assertive, thus, this study should be supported by a prospective study to compare these 2 groups with larger sample sizes.

The enhancement of the efficiency of colposcopy should focus on improving the detectability of precancerous lesions. Given that this study compared the effectiveness of acetic acid concentration in colposcopy diagnostics, it can be considered a leading study in the field. Therefore, optimal acetic acid concentration should be determined to achieve the best efficiency in colposcopy diagnosis by conducting comprehensive studies with more cases and, most importantly, prospective randomized controlled trials.

## Author contribution

**Conceptualization:** Alpay Yilmaz, Aysegul Gulbahar.

**Data curation:** Alpay Yilmaz, Aysegul Gulbahar.

**Formal analysis:** Alpay Yilmaz, Aysegul Gulbahar.

**Investigation:** Alpay Yilmaz, Aysegul Gulbahar.

**Methodology:** Alpay Yilmaz, Aysegul Gulbahar.

**Resources:** Alpay Yilmaz, Serhat Sen.

**Supervision:** Alpay Yilmaz.

**Writing – original draft:** Alpay Yilmaz, Aysegul Gulbahar.

**Writing – review & editing:** Alpay Yilmaz, Aysegul Gulbahar, Serhat Sen.

## Supplementary Material


