# Supplementary material for: Comparison of the efficacy of 5% versus 8% acetic acid concentrations for detecting premalignant and malignant lesions in colposcopy
Source: Medicine (Baltimore). 2023 Dec 15;102(50):e36341. doi: 10.1097/MD.0000000000036341 (PMC10727527; doi:10.1097/MD.0000000000036341)

Supplemental Digital Content Legends

Figure 1. Figure that illustrates the colposcopic images of the cervix after 5% and 8% acetic acid application.

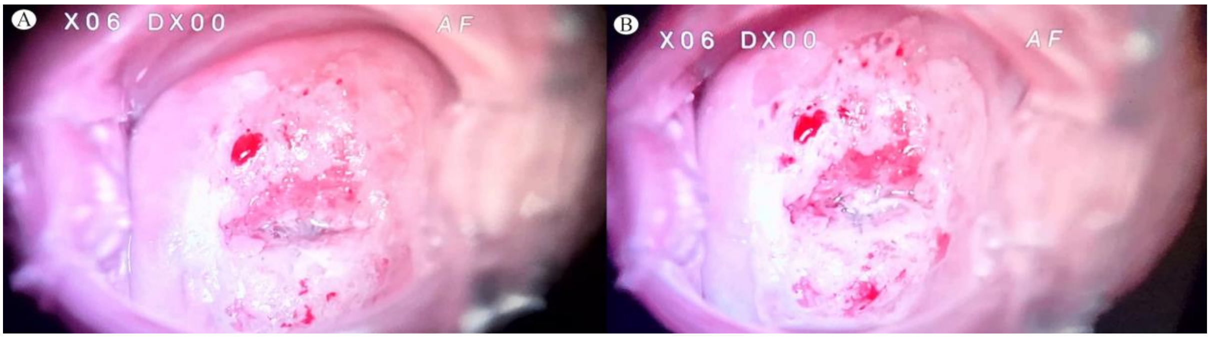

Supplement: Supplementary file 1 [file medi-102-e36341-s001.pdf]
